# Cyclic Strain and Electrical Co-stimulation Improve Neural Differentiation of Marrow-Derived Mesenchymal Stem Cells

**DOI:** 10.3389/fcell.2021.624755

**Published:** 2021-05-11

**Authors:** Hong Cheng, Yan Huang, Wei Chen, Jifei Che, Taidong Liu, Jing Na, Ruojin Wang, Yubo Fan

**Affiliations:** ^1^Beijing Advanced Innovation Center for Biomedical Engineering, Key Laboratory for Biomechanics and Mechanobiology of Chinese Education Ministry, School of Biological Science and Medical Engineering, Beihang University, Beijing, China; ^2^School of Engineering Medicine, Beihang University, Beijing, China

**Keywords:** mesenchymal stem cells, strain, electrical stimulation, neuron, differentiation

## Abstract

The current study investigated the combinatorial effect of cyclic strain and electrical stimulation on neural differentiation potential of rat bone marrow-derived mesenchymal stem cells (BMSCs) under epidermal growth factor (EGF) and fibroblast growth factor 2 (FGF2) inductions *in vitro*. We developed a prototype device which can provide cyclic strain and electrical signal synchronously. Using this system, we demonstrated that cyclic strain and electrical co-stimulation promote the differentiation of BMCSs into neural cells with more branches and longer neurites than strain or electrical stimulation alone. Strain and electrical co-stimulation can also induce a higher expression of neural markers in terms of transcription and protein level. Neurotrophic factors and the intracellular cyclic AMP (cAMP) are also upregulated with co-stimulation. Importantly, the co-stimulation further enhances the calcium influx of neural differentiated BMSCs when responding to acetylcholine and potassium chloride (KCl). Finally, the phosphorylation of extracellular-signal-regulated kinase (ERK) 1 and 2 and protein kinase B (AKT) was elevated under co-stimulation treatment. The present work suggests a synergistic effect of the combination of cyclic strain and electrical stimulation on BMSC neuronal differentiation and provides an alternative approach to physically manipulate stem cell differentiation into mature and functional neural cells *in vitro*.

## Introduction

Traumatic nervous system injuries, stroke, and many neurological disorders are characterized by the loss of neuronal functions. The damaged neural tissue rarely recovers spontaneously due to extremely low endogenous regenerative capacity and poor migrating ability of the neural stem cells. Stem-cell-mediated therapy has shown a great preclinical potential for neural injury and degenerative diseases. Mesenchymal stem cells (MSCs) have been widely used as a cell therapy to treat various diseases including bone diseases, cardiovascular diseases, autoimmune diseases, and inflammatory diseases (Shafei et al., 2017; Molendijk et al., 2018; Su et al., 2018; Yan et al., 2018). It is well established that MSCs have the capability to differentiate into several cell types, such as osteoblasts, chondrocytes, neural cells, hepatocytes, lung cells, and vascular endothelial cells (Chen et al., 2004; Tropel et al., 2006; Aurich et al., 2009; Jang et al., 2010). Previous work has demonstrated that MSCs can differentiate into neural-like cells under various conditions *in vitro* and *in vivo* (Deng et al., 2001; Cho et al., 2005; Yang et al., 2008). Furthermore, animal experiments showed that MSC-differentiated neuronal cells are beneficial for neuronal regeneration (Brazelton et al., 2000; Takizawa, 2003; Mimura et al., 2005; Bahat-Stroomza et al., 2009; Hayase et al., 2009).

Many treatments, including chemical compounds, growth factors, and genetic manipulation, have been adopted to improve BMSC neural differentiation (Deng et al., 2001; Cho et al., 2005; Yang et al., 2008). However, it suggested that morphological changes and a modest increase of gene expression levels for neural markers promoted by chemical induction were not real neurogenesis but merely cellular toxicity or cytoskeletal changes (Bertani et al., 2005). A growing number of bioengineering strategies such as cell culture biomaterials, mechanical force, and electrical field have been explored to evaluate the potential cues on the differentiation of MSCs into neural lineages. Studies have demonstrated that electrical stimulation plays a key part in broad biological activities, including proliferation, differentiation, and activation of intracellular pathways of various cell types (Schmidt et al., 1997; Sheikh et al., 2013; Yuan et al., 2014; Taghian et al., 2015). Specifically, electric field has been reported to be able to direct neural cell migration and neurite growth as well as promote neural stem cell proliferation and differentiation (Pan and Borgens, 2012; Babona-Pilipos et al., 2015; Pires et al., 2015; Petrella et al., 2018). In addition, electric field stimulation could repair the injury of neurons by increasing Netrin-1 and its receptor expression (Liu et al., 2018). Clinical applications of low-frequency electrical stimulation showed benefits of improved nerve regeneration and functional recovery (Gordon et al., 2009). On the other hand, native stem cells respond to dynamic local mechanical forces which show important regulatory roles in cell proliferation, metabolism, differentiation fates, and survival (Vining and Mooney, 2017; Romani et al., 2019). Accruing evidence showed that mechanical and physical cues, such as fluid shear stress, static stretch, and magnetic forces, can also contribute to stem cell fate determination (Clause et al., 2010; Marycz et al., 2016; Vining and Mooney, 2017). A recent study has revealed that extracellular physical cues could transduce into intracellular force to control the intestinal organoid growth and development through Wnt/β-catenin signaling (Li et al., 2020). Particularly, stretch could stimulate neuron growth (Loverde and Pfister, 2015; Breau and Schneider-Maunoury, 2017), axon growth (De Vincentiis et al., 2020), and neurite outgrowth (Higgins et al., 2013; Kampanis et al., 2020). Moreover, we have reported that fluid shear stimulation could boost BMSC differentiation into endothelial cells and cardiomyocyte-like cells (Bai et al., 2010; Huang et al., 2010).

In the present study, we examined the effect of the association of mechanical strain with electrical stimulation on BMSC neural differentiation, which was not observed under each individual stimulation. Cells were seeded on elastic silicone membranes and subjected to cyclic uniaxial stretching and/or electrical stimulation. Morphological characters, neuronal biomarker expression level, and calcium influx were evaluated under different treatments. Besides, transcriptome analysis was applied to elucidate the potential biological processes and signaling pathways of electric fields and strain co-stimulation-directed neuron differentiation. We proposed that the combined mechanical and electrical stimulation will potentially improve BMSC differentiation into neural cells.

## Materials and Methods

### BMSC Culture

Primary BMSCs were isolated from the femurs and tibias from 4-week-old male Sprague-Dawley rats (Beijing Vital River Laboratory Animal Technology Co., Ltd, Beijing, China) by Percoll technique (Pharmacia, Uppsala, Sweden) as previously described (Huang et al., 2010). Isolated cells were seeded in 10 cm plastic culture dish and cultured in Dulbecco’s modified Eagle medium-low glucose (DMEM-LG; Gibco, Grand Island, NY) containing 10% fetal bovine serum (FBS, Gibco). Non-adherent cells were removed after seeding for 3 days, and the medium was refreshed every 3 days. Cells were passaged when the cells reached 90% confluency by trypsin digestion, and cells used for all experiments were between passages 2–4. Isolated cells were confirmed by our lab that they expressed mesenchymal cell markers CD29, CD44, CD90, CD105, CD106, and CD166 and negative for CD34, CD45, and HLA-DR by flow cytometry analysis (Huang et al., 2010). Isolated cells also showed the multipotency to differentiate into osteoblasts (Li et al., 2014), endothelial cell (Bai et al., 2010), and cardiomyocyte-like lineage (Huang et al., 2012) in our previous studies.

### Device

**FIGURE 1:**
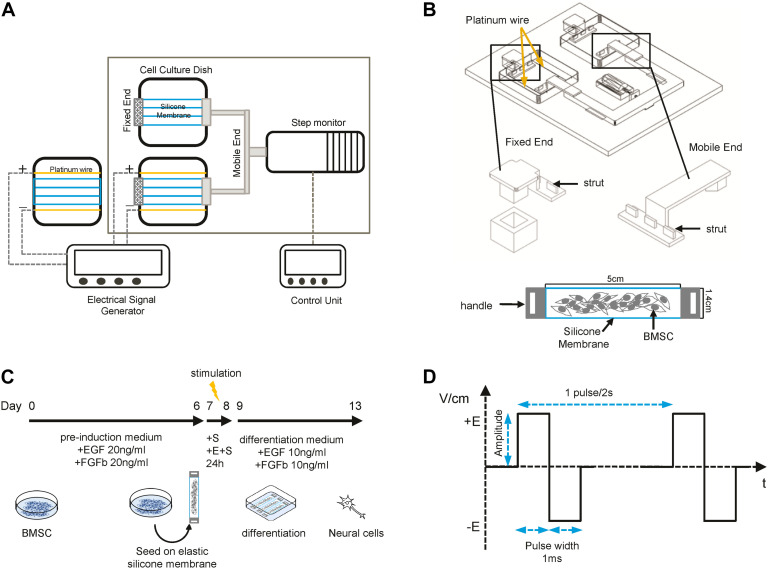
Sketch of the device and differentiation process. **(A)** Schematic representation of the stretching and electrical field (EF) stimulation device. **(B)** Detailed structure of fixed and mobile ends and the elastic silicone membrane. **(C)** Schematic overview of the BMSC neural differentiation process. **(D)** The pulsed biphasic electrical signal.

A self-designed device which could provide cyclic strain and pulsed biphasic electrical field (EF) stimulation was developed as shown in Figures 1A,B. The apparatus consisted of a step motor controlled by a motor driver and a signal amplifier, an alternating current signal generator, and a culture chamber with a transparent lid. Inside the culture chamber, there were two quadrate plastic culture plates, two fixed ends, and two mobile ends which can move forward and back under the control of the step motor driver. There were three struts on each end. BMSCs were seeded at the density of 2 × 10e4/cm^2^ on pieces of elastic silicone membrane (USP class VI silicone, durometer 40, elastic modulus 7.7 GPa) with two handles. The strain was created by the stretching and shrinking of the elastic silicone membrane after putting the handles of the membrane onto the struts on fixed and mobile ends. To generate the bidirectional pulse current, two platinic wires were placed in the plate and connected to the alternating current signal generator. The electrical field was 1 V/cm, 0.5 Hz (Figure 1D). The system was kept inside an incubator and sterilized by UV light for 30 min. Parallel static control cells were cultured on the silicone membrane without electrical or strain stimulation.

### Neural Differentiation and Treatment

Cells were pretreated with a preinduction medium [DMEM-LG supplemented with 2% B27 (Gibco), 20 ng/ml fibroblast growth factor 2 (FGF2, A sigma), and 20 ng/ml epidermal growth factor (EGF, sigma)] for 7 days and then seeded on the elastic silicone membrane which was precoated with 0.01% poly-L-lysine (sigma). Then, the membranes were cultured under static and dynamic conditions with or without ES for 24 h. The membranes were then put in a plastic dish, and the medium was changed into differentiation medium (DMEM-LG medium supplemented with 10% FBS, 2% B27, 10 ng/ml FGF2, 10 ng/ml EGF, 100 U/ml penicillin, and 100 mg/ml streptomycin). Cells were differentiated for another 5 days and then harvested for qPCR, immunocytochemistry, and other assays (Figure 1C).

### RNA Extraction and Quantitative RT-PCR

Total RNA isolation from cells under different treatments was performed with the Qiagen RNeasy Plus Mini Kit (Qiagen, Hilden, Germany). cDNA was synthesized from total RNA samples using a Reverse Transcription Kit (TaKaRa, Kyoto, Japan). The forward and reverse primers used for quantitative RT-PCR were synthesized by Sangon Biotech (Shanghai, China); the sequences are listed in Table 1. Also, qPCR was performed on an Applied Biosystems ViiA^TM^ 7 Real-Time PCR System (Thermo Fisher Scientific, United States). Three replicas were performed in the qPCR analysis and the relative gene expression compared to the housekeeping gene GAPDH. Data from at least three independent experiments were collected.

**TABLE 1:** The primers for RT-PCR.

Gene	Sense 5→3	Antisense 5→3	Size (bp)
NSE	CCGGGTCAAGACGCTAGAAGA	CTCCAGCTCTTCCGCAAGGTTGT	196
β-Tubulin III	GTCCGCCTGCCTCTTCGTCTCTA	GGCCCCTATCTGGTTGCCGCACT	93
MAP2	CAAACGTCATTACTTTACAACTTGA	CAGCTGCCTCTGTGAGTGAG	122
NT-3	CTTCTGCCACGATCTTAC	AACATCTACCATCTGCTTG	197
NT-4	CTAATGTGTGACTCTGCTAAC	GATACGGTGCTCAGGATAG	180
BDNF	GCGGCAGATAAAAAGACTGC	GCCAGCCAATTCTCTTTTTG	238
GAPDH	GGTGTGAACGGATTTGGCCGTAT	CTCAGCACCAGCGTCACCCCATT	262

### RNA Sequencing Analysis

Total RNA sequencing was performed at Novogene Bioinformatics Technology Co. Ltd. (Beijing, China). HISAT2-2.1.0, StringTie-1.3.5, and DEseq were used to select the differentially expressed genes. Genes with adjusted *p* < 0.05 and log2(Fold Change) > 1 were screened out as significantly differentially expressed. Gene Ontology enrichment analysis was performed using the DAVID online tool. GO terms with corrected *p* < 0.05 and a fold change > 1.5 were considered to be significantly enriched by differentially expressed genes. The pathway enrichment analysis was based on the latest Kyoto Encyclopedia of Genes and Genomes (KEGG) database. The Benjamini and Bonferroni approaches were used to control the false discovery rate.

### Immunocytochemistry and Image Analysis

Cells were fixed with 4% paraformaldehyde (PFA; Sigma-Aldrich) for 10 min at room temperature (RT), triple rinsed with phosphate-buffered saline (PBS), and then permeabilized with 0.1% Triton X-100 for 10 min, followed by blocking with 5% BSA for 1 h at RT. Samples were incubated with primary antibodies anti-Nestin antibody (Abcam, cat# ab134017, diluted at 1:10,000) and anti-neuron-specific class III beta-tubulin (Abcam, cat#ab52623 diluted at 1:1,000), then washed three times with PBS, stained with secondary antibodies for 1 h at RT. Secondary antibodies included rabbit anti-chicken IgY H&L FITC (Abcam, cat#ab6749, diluted at 1:1,000) and R-Phycoerythrin AffiniPure F(ab′)_2_ Fragment Goat Anti-Rat IgG (H + L) (Jackson ImmunoResearch, cat#112-116-143, diluted at 1: 200). 4′,6-Diamidino-2-phenylindole (DAPI, Dojindo, cat#28718-90-3) was used for nuclear staining. Rhodamine phalloidin (Thermo Fisher Scientific, cat#R415, 1: 200) was used for staining actin filaments. Confocal images were photographed using Leica DMI4000B.

The morphologic parameters were measured from images captured by the Olympus inverted microscope equipped with the Olympus digital camera DXM-1200 (Nikon Canada) and confocal microscope (Leica, TCS SPE). All images were analyzed by ImageJ package, Fiji. The neurite length was analyzed by Fiji with NeuronJ plugin (Pemberton et al., 2018), and lengths of the longest neurite for 44 cells per condition were used for statistical analysis.

### Flow Cytometry Analysis

Cells were harvested and fixed with fixation/permeabilization solution (BD Pharmingen^TM^) for 10 min at RT, washed with 1 × Perm/Wash Buffer (BD Pharmingen^TM^), and then resuspended in 1 × Perm/Wash Buffer (2% BSA in PBS). 1 × 10e5 cells/well were incubated with first antibodies (anti-Nestin antibody, anti-III beta-tubulin) for 30 min at RT followed by twice washing steps with PBS. Cells were resuspended in 1 × Perm/Wash Buffer and incubated with relative fluorochrome-labeled second antibodies [rabbit anti-chicken IgY H&L FITC, R-Phycoerythrin AffiniPure F(ab′)_2_ Fragment Goat Anti-Rat IgG (H + L)] for 30 min at RT. Cells were analyzed by flow cytometry using a BD FACSCelesta and FlowJo software (BD Biosciences, Heidelberg, Germany).

### Measurement of cAMP and Phosphorylation of ERK

Quantification of cAMP in BMSC-derived neural cells after stimulation was carried out using a commercial kit (LANCE^®^ Ultra cAMP Kit). After the strain and/or electrical stimulation, the differentiated cells were collected and seeding at 1,000 cells per well in a white OptiPlate^TM^-384 microplate and then followed the manufacturer’s guidance. The time-resolved fluorescence resonance energy transfer (TR-FRET) signal was measured on an EnVision^®^ Multilabel reader (PerkinElmer, United States). The cAMP level was calculated according to the standard curve.

The phosphorylation of ERK and AKT was detected by AlphaLISA^®^ SureFire^®^ Ultra^TM^ p-ERK 1/2 (Thr202/Tyr204) assay kit and AlphaLISA SureFire Ultra p-AKT1/2/3 (Thr308) Assay Kit, respectively (PerkinElmer, United States).

### Live Cell Calcium Test

After differentiation, BMSC-derived neural cells were collected for calcium test using the fluorometric imaging plate reader (FLIPR Tetra, Molecular Devices, United Kingdom). Cells were seeded into 384-well plates with the density of 10,000 cells/well (25 μL) and cultured overnight before incubating with an equal volume of FILIPR Calcium 6 indicator (FLIPR Calcium 6 Assay Kits, Molecular Devices) in Hank’s balanced salt solution (HBSS with 20 mM HEPES, pH 7.4) for 2 h at 37°C. Response signals (relative fluorescence units, RFU) were traced during 190 s when the stimuli acetylcholine (final concentration 0.1 mM) and KCl (final concentration 45 mM) were added automatically using the FLIPR instrument. To enable comparison, baseline was subtracted from response signals. Moreover, the peak amplitude was calculated by maximal–minimal signal.

### Statistical Analysis

Cells for all experiments were isolated from at least three donors of rats, and all data were collected from independent isolations. Statistical analysis was performed using GraphPad Prism v.8.0 software (GraphPad Inc., San Diego, CA, United States). Graphed data were presented as mean ± standard deviation from at least three independent biological replicates. Groups were compared using Mann–Whitney Test *t*-tests and one-way analysis of variance (ANOVA) as appropriate. ^∗^*p* < 0.05 and ^∗∗^*p* < 0.01 were considered statistically significant.

## Results

### Cell Alignment Under Cyclic Strain and Electrical Stimulation

**FIGURE 2:**
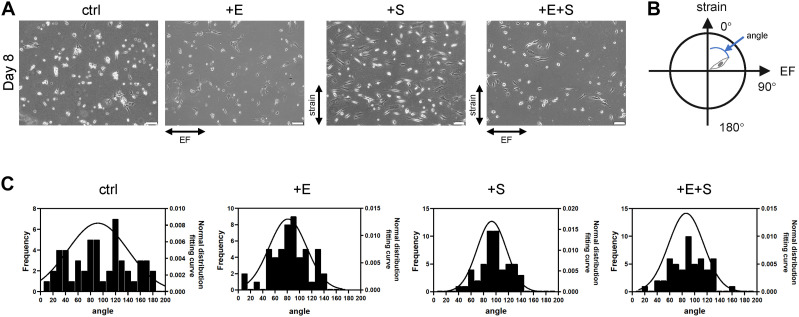
BMSC reorientation under cyclical strain and electrical field stimulation. **(A)** The change of cellular orientation under static control (ctrl), electrical stimulation (+E), strain (+S), and co-stimulation (+E + S). Scale bar, 100 μm. The directions of strain and electrical field were indicated by arrows. **(B)** Schematic illustration indicates cell angle. The vertical upward direction was defined as 0°, and the horizontal right direction was defined as 90°. **(C)** Distribution of cellular orientation. The line was the normal distribution fitting curve.

The rat BMSCs were preinduced for 7 days, and then pyramidal-shaped cell bodies and extended short neurites, reminiscent of dendrites, could be identified. To test the combinatorial effect of strain and EF, cells were subjected to cyclic strain (5% elongation, 0.5 Hz, + S), EF (1 V/cm, 0.5 Hz, + E), and co-stimulation (+ E + S) for 24 h before changing to a differentiation medium. Under strain and electrical stimulation, cells showed orientation change and alignment (Figure 2A). The cells in static control culture showed a random orientation. Cells under strain became oriented away from (perpendicular to) the direction of cyclic stretch, and cells under electrical stimulation aligned themselves with the direction of electrical stimulation. Some cells detached from the membranes during strain or electrical stimulation, and a few more cells detached and died under co-stimulation, but the remaining cells were still in good condition (Supplementary Figure 1). To quantify the cell orientation (Figure 2B), angles of 52 cells for each treatment were measured. The cell orientation distribution was analyzed by the cell frequency in each direction (Figure 2C). Cells under strain, electrical stimulation, and co-stimulation showed an increase in the frequency of cells oriented at angles near 90°.

### Cyclic Strain and Electrical Co-stimulation Enhanced Neural Morphological Change

**FIGURE 3:**
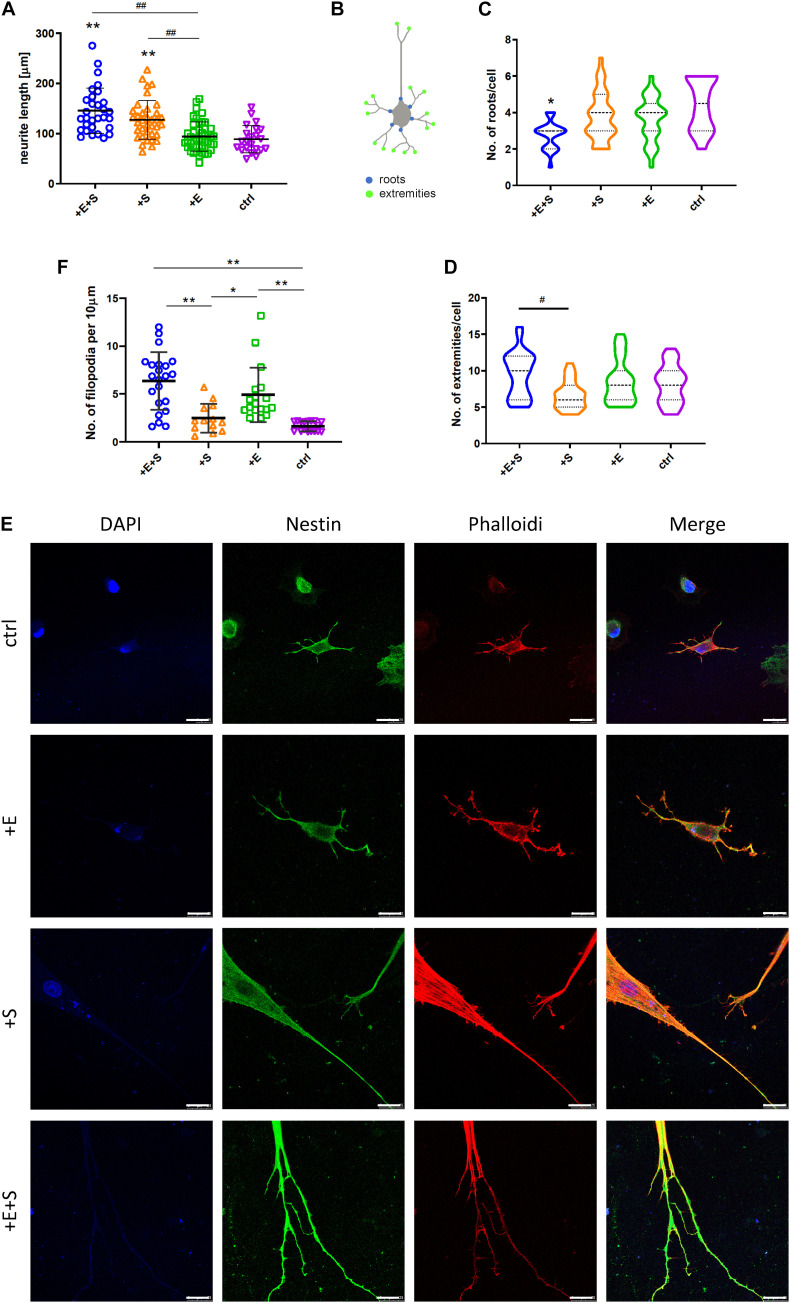
BMSCs’ morphologic change under cyclical strain and electrical field stimulation. **(A)** Co-stimulation (+E +S) and strain (+S) significantly elongated neurites compared with static control (ctrl) (**p < 0.01) and electrical stimulation (+ E) (^##^*p* < 0.01, ANOVA). **(B)** Diagram of the roots and extremities of neurites. The numbers of roots **(C)** and extremities **(D)** of neurites under each treatment were counted manually from four independent experiments. Values are mean ± SD. **(E)** Immunocytochemistry detecting actin filament (red), nestin (green), and nucleus (blue) expression in rBMSCs under treatments (scale bar = 25 μm). **(F)** Density quantification of filopodia under each treatment. The number of filopodia per 10 μm of neurite was used to calculate the filopodia density (*p < 0.05, **p < 0.01, ANOVA). ^#^*p* < 0.05.

After another 5 days of differentiation, cells had typical morphological features of neurons, such as extending and branching processes. Morphology of cells was evaluated by the following parameters: the longest length of neurites and the number of the root and extremity of neurites (Figure 3B). Cells under strain alone and co-stimulation induced significantly longer neurites compared with electrical stimulation and static control (Figure 3A). The cyclic strain plus electrical stimulation could further increase the length than electrical treatment alone, indicating the enhanced impact of strain on neurite growth. Although co-stimulation induced additional increase in neurite length compared with strain alone, there was no significant difference. In contrast to neurite length, there were few neurite roots from cells under co-stimulation than under static control (Figure 3C); however, the extremity index was similar under different conditions except for the lower-extremity index under strain stimulation compared with co-stimulation (Figure 3D). Thin, hair-like filopodia can be seen along the neurites (Figure 3E). Compared with the strain and control groups, the filopodia density (the number of filopodia per 10 μm neurite length) was significantly higher in electrical stimulation and co-stimulation conditions (Figure 3F).

### Cyclic Strain and Electrical Co-stimulation Increase Neural Cell Marker Expression

**FIGURE 4:**
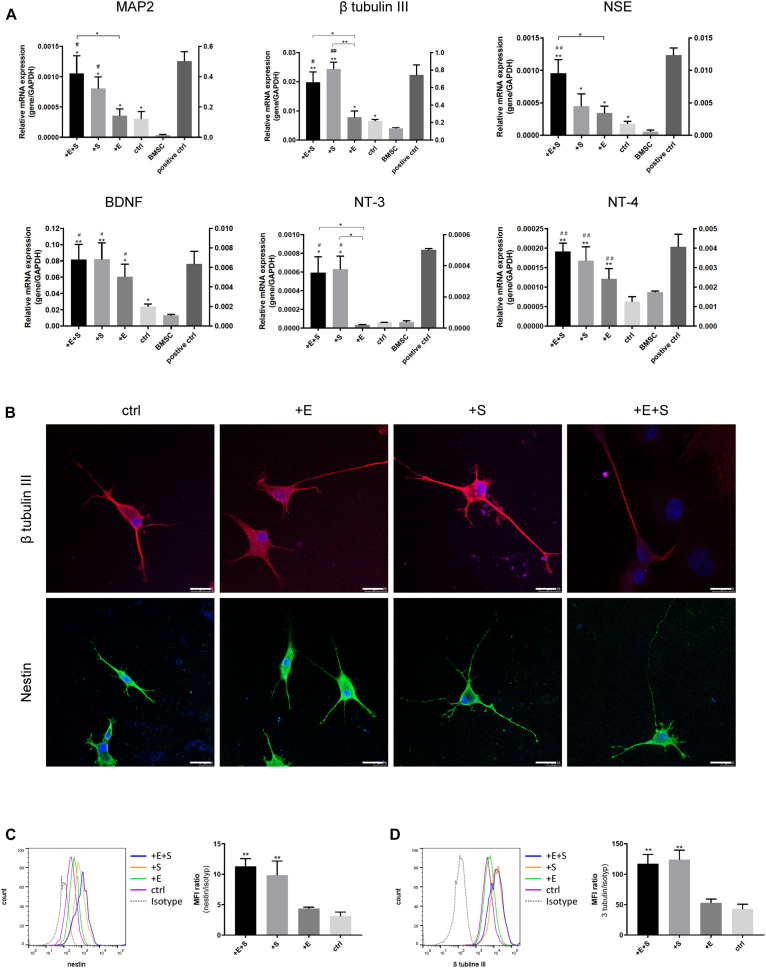
Effects of the strain and electrical stimulation on the neural related gene expressions of BMSCs. **(A)** BMSCs were induced by the neural differentiated medium under static conditions (ctrl) or under cyclic strain (+S, 5% elongation, 0.5 Hz), under electrical stimulation (+E, 1 V/cm, 0.5 Hz), and under co-stimulation (+ E + S) for 24 h. Gene expression of MAP2, β-tubulin III, NSE, BDNF, NT-3, and NT-4 on day 13 was analyzed by real-time RT-PCR and normalized to GAPDH. Normal neonatal rat neurons were used as positive control. Results are shown as mean ± SD (*N* = 4). *p < 0.05, **p < 0.01 compared to the BMSC, ^#^*p* < 0.05, ^##^*p* < 0.01 compared to the static control. **(B)** Representative immunostaining images of neural differentiated BMSCs under treatments. Immunocytochemistry detecting β tubulin III (red) and nestin (green) expressions in BMSCs with DAPI (blue) under different treatments (scale bar = 25 μm). Representative flow cytometry histograms showing the protein expression of β tubulin III (**C** left) and nestin (**D** left) and statistical analysis of β tubulin III (**C** right) and nestin (**D** right) expression level under treatments (*n* = 3, **p < 0.01).

The influence of cyclic strain and electrical co-stimulation on gene expression of neural cell markers and neurotrophins involved in neural development was analyzed by qPCR. Compared to BMSC or electrical stimulation alone, co-stimulation induced a significant upregulation of Microtubule Associated Protein 2 (MAP2), β tubulin III, neuron-specific enolase (NSE) as well as neurotrophins, NT-3, NT-4, and brain-derived neurotrophic factor (BDNF) (Figure 4A). BMSCs differentiated into neural cells were further confirmed by positive staining of the immature neuron marker Nestin, and the immature and mature neuron marker β tubulin III (Figure 4B). The flow cytometry data confirmed that under strain or co-stimulation, the nestin and β tubulin III protein expression levels were significantly increased compared to static control (Figures 4C,D).

### Cyclic Strain and Electrical Co-stimulation Enhanced the Neural Differentiation

**FIGURE 5:**
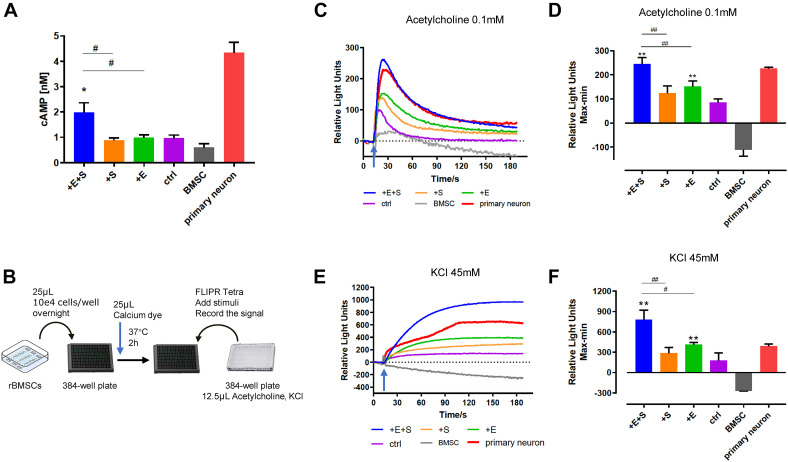
Electrical field and cyclical stretch co-stimulation enhanced the rBMSC-derived neural cell function. **(A)** cAMP level in differentiated cells under static condition (ctrl), strain (+S), electrical stimulation (+E), and co-stimulation (+E +S) (*n* = 9). **(B)** Schematic of the calcium test process. Calcium signaling triggered (arrows indicate the time point of adding inducer) by acetylcholine (0.1 mM) **(C,D)** and KCl (45 mM) **(E,F)**. The primary neurons cultured *in vitro* for 7 days were used as a positive control, and the undifferentiated BMSCs were the negative control. Representative tracings of calcium signal record by FLIPR after adding acetylcholine **(C)** and KCl **(E)**. Statistical analysis of the peak amplitude **(D,F)**. *p < 0.05, **p < 0.01 (compared with static control), ^#^*p* < 0.05, ^##^*p* < 0.01 (ANOVA, *n* = 5).

It is well established that cyclic AMP (cAMP) signaling cascade plays an important role in neuronal differentiation, axonal guidance, neurite outgrowth, and neuron maturation (Cai et al., 2002; Fujioka et al., 2004; Aglah et al., 2008). As shown in Figure 5A, the cAMP levels under all the treatments increased after being differentiated from BMSCs. Specifically, for the co-stimulation, the level of intracellular cAMP was doubled compared to that of electrical or strain simulation alone.

Calcium signals are known to be important regulators of neurite outgrowth as well as a charge carrier. The calcium change was detected by the FLIPR system. Figures 5C,E show a representative calcium tracing signal when differentiating BMSCs treated with 0.1 mM acetylcholine and 45 mM KCl. Electrical stimulation and co-stimulation triggered higher calcium influx induced by acetylcholine (Figure 5D) and KCl (Figure 5F) than static control. Moreover, cells produced a significant higher calcium signal under co-stimulation than strain or electrical treatment alone (Figures 5D,F).

### Cyclic Strain and Electrical Co-stimulation Altered mRNA Expression

**FIGURE 6:**
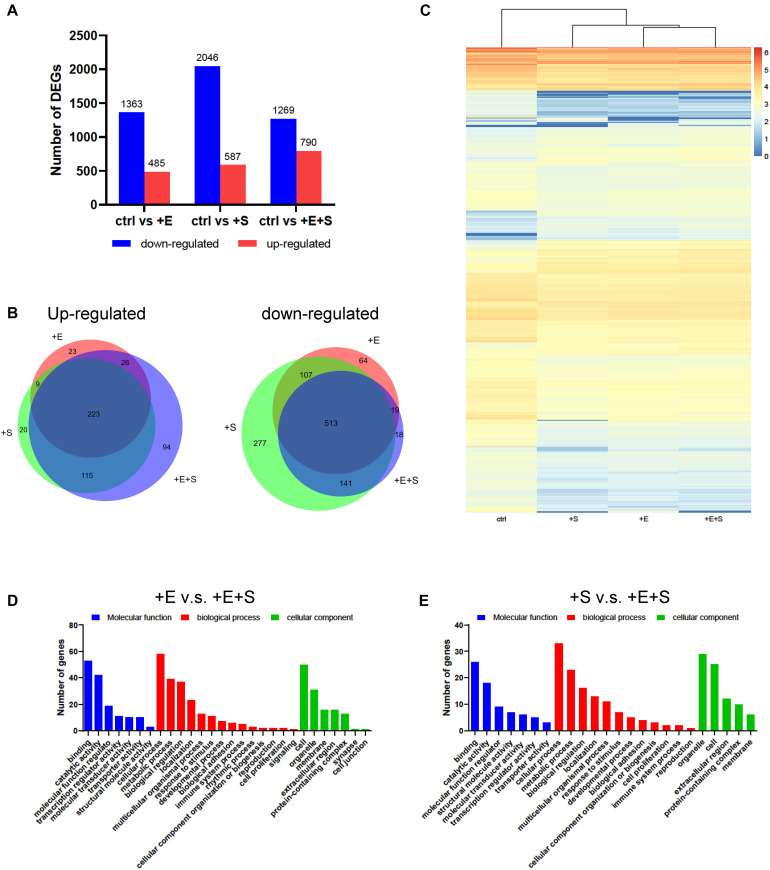
Changes in gene expression profiles of neural differentiated BMSCs under different stimulations. **(A)** Numbers of DEGs compared with only EGF and FGF2 induction with EF and/or stain treatments. **(B)** Venn diagram showed the overlap genes among different treatments. **(C)** Heat map diagrams showed the relative expression levels of total DEGs under different stimulations. **(D)** DEGs between EF and co-stimulation. **(E)** DEGs between strain and co-stimulation.

We examined the transcriptional changes via RNA sequencing for differentiated cells under strain and/or electrical stimulation and under control conditions. In total, 985, 1,406, and 1,150 DEGs displayed a differential expression between electrical stimulation, strain, and co-stimulation groups compared to no treatment control, respectively (Figure 6A). Ninety-four upregulated genes and 18 downregulated genes were screened out in the electrical and strain co-stimulation groups (Figure 6B). Hierarchical clustering shows a general overview of the expression pattern among samples (Figure 6C).

The enriched genes for the electrical stimulation or strain vs. co-stimulation comparison are summarized in three main GO categories (molecular function, biological process, cellular component). As shown in Figures 6D,E, the genes’ differential expression in both electrical stimulation vs. co-stimulation and strain vs. co-stimulation comparison is highly enriched for “binding,” “catalytic activity,” “cellular process,” “metabolic process,” and “biological regulation.”

### Cyclic Strain and Electrical Co-stimulation Activated Pathway Analysis

**FIGURE 7:**
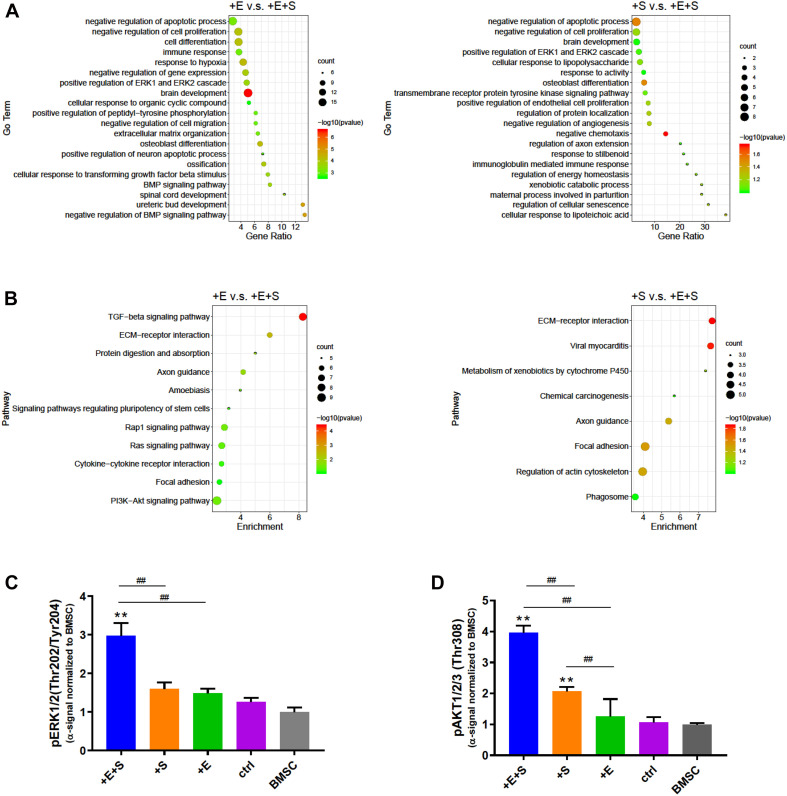
Signaling pathway evaluation under different treatments. **(A)** Go term analysis between EF vs. co-stimulation and strain vs. co-stimulation. **(B)** KEGG pathway enrichment analysis of EF or strain vs. co-stimulation. Phosphorylation of ERK1/2 (Thr202/Tyr204) **(C)** and AKT1/2/3 (Thr308) **(D)** was detected by alpha screening assay. The alpha signal was normalized to that of BMSC (*n* = 6, **p < 0.01 compared with static control, ^##^*p* < 0.01, ANOVA).

We next determined the strain and electrical co-stimulation effect on neural differentiation. Comparing EF and strain treatment only, the co-stimulation enriched GO terms are involved in the positive regulation of the ERK1 and ERK2 cascade, negative regulation of cell proliferation, and brain development (Figure 7A). In the KEGG pathway analysis, the DEGs are found to be enriched in focal adhesion, ECM–receptor interaction, and axon guidance in both electrical stimulation vs. co-stimulation and strain vs. co-stimulation comparison (Figure 7B). Furthermore, the PI3K-AKT signaling pathway is the highest pathway count in electrical stimulation vs. co-stimulation.

To confirm the signaling pathway involved under strain and electrical co-stimulated conditions during neural differentiation, we examined the phosphorylation level of ERK and AKT. Consistent with GO and KEGG pathway analyses, co-stimulation significantly increases the level of phospho-ERK and phospho-AKT than strain and electrical stimulation alone (Figures 7C,D). Moreover, the level of phospho-AKT in strained cells is also significantly higher than that in no treatment control cells. These data suggests that strain and electrical co-stimulation could contribute significantly to the activation of ERK and AKT pathways in BMSC neuronal differentiation processes.

### Protein and Protein Interaction Analysis Under Strain and Electrical Co-stimulation

**FIGURE 8:**
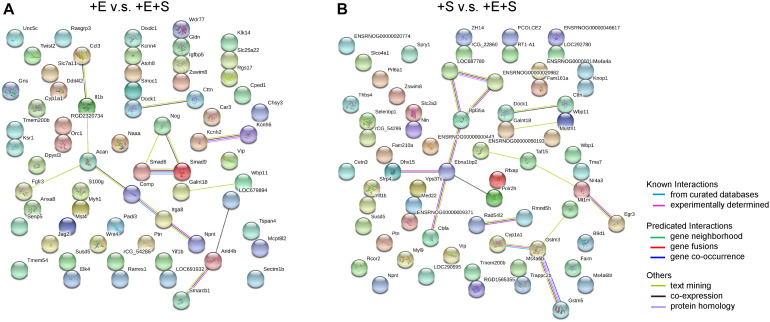
Protein–protein interaction networks by STRING search tool. The up- and downregulated mRNAs (fold change > 1, *p* < 0.05) network between electrical stimulation with co-stimulation **(A)** and strain with co-stimulation **(B)**. Analysis by STRING based on protein–protein interactions. The high confidence score (0.7) was adopted to evaluate the protein interactions for the differentially expressed genes.

To further investigate the differentially expressed genes at the protein level in the differentiation process of BMSCs under co-stimulation, a biological database, search tool/STRING, was used to filter functional genes. The protein–protein interaction was analyzed online to provide an intuitive network for the functional properties of proteins. The STRING analysis shows that in the + E vs. + E + S comparison group, genes for potassium voltage-gated channel subfamily H member 2 and 6 (Kcnh2, Kcnh6) are functionally linked. Besides, nodes Comp, Itga8, and Npnt and nodes Smad6, Smad9, and Nog are linked, respectively (Figure 8A). Comp is an extracellular matrix protein, and NPNT binds to integrin alpha-8/beta-1, suggesting a key role in regulating cell adhesion, spreading, and survival. Smad6 and Smad9 encode proteins that are signal transducers and transcriptional modulators which are involved in numerous signaling pathways. Smad6 is highly expressed in mature neurons and can promote cells that differentiate into mature neurons (Hazen et al., 2011; Xie et al., 2011). The Nog gene-encoded protein can regulate neural crest formation. In the + S vs. + E + S comparison group, the most connected protein nodes are Cyp1a1, Gstm3, Gstm5, and Mt1m (Figure 8B), which are essential for cell metabolism. Cyp1a1 encodes the cytochrome P450 enzyme. Gstm (Glutathione S-Transferase Mu)3 and 5 are related pathways which are glutathione metabolism and platinum drug resistance. Mt1m encodes a well-known metallothionein.

## Discussion

Identify a method that is capable of promoting neural cells different from stem cells is of great interest in treating and repairing nerve damage. A great number of previous investigations have suggested that BMSCs possess the capability of differentiating into neural cells when exposed to defined chemical reagents, trophic factors, or genetic manipulation. Besides, a few reports have investigated that physical cues, such as electrical or mechanical stimulation, could enhance cell neural differentiation (Rajnicek et al., 2006; Thrivikraman et al., 2014; Pires et al., 2015). Our current study demonstrated that electrical and cyclic uniaxial stretching co-stimulation together with EGF and FGF2 could promote BMSC neural differentiation, neurite outgrowth, and active ERK1/2, AKT signaling pathways.

In this study, we used a self-designed device to provide cyclic strain (5%, 0.5 Hz) and electrical field (1 V/cm, 0.5 Hz) simultaneously. Consistent with previous studies suggesting that stretch and EF can regulate cell orientation (Neidlinger-Wilke et al., 2001; Haq et al., 2006; Arocena et al., 2010; Tang-Schomer, 2018), we observed cell reorientation and alignment with the direction of the loading axis and electrical field. In addition, cyclic strain and co-stimulation induced longer neurites than did electrical stimulation and static control. Similar findings have been reported that cyclic stretch alone can induce neurite outgrowth of SH-SY5Y (a human neuroblastoma cell line cell, 10%, 0.25 Hz) and PC12 cells (a rat pheochromocytoma cell line, 4%, 1 Hz or 16%, 0.1 Hz) (Haq et al., 2006; Higgins et al., 2013) and trigger human MSCs to differentiate into neuron-like cells at very low amplitude loading (0.5%, 0.5 Hz) (Leong et al., 2012). Moreover, stretch is also found to stimulate neurite growth of mature neurons. Ten percent cyclic stretch of nerve explants at 0.5 Hz enhanced neurite outgrowth of neurons from rat dorsal root ganglia (Kampanis et al., 2020), and 10 pN of stretch could enhance axon growth and branching (De Vincentiis et al., 2020). However, the conclusions of the amplitude of cyclic strain that can induce neurite outgrowth or neural differentiation are different from these studies. This may be due to the different cell types and the degree of neural cell maturity.

From our study, cyclic strain and electrical co-stimulation showed effects not only on neurite outgrowth but also on neurite branching and filopodia density. There was a significant decrease in the number of roots of neurite under co-stimulation compared with static control, but not with strain or electrical treatment alone. This correlates with a study by Feng et al. (2016) reporting that stretch could reduce the number of neurites because mechanical tension initiated major neurites to grow preferentially near the cell poles closest to the source of tension. In addition, the alternating EF also demonstrated a robust directing effect on axon alignment (Tang-Schomer, 2018). The hypothesis is that stretch and EF have synergetic effects on cell alignment which may last for a longer time than strain or EF treatment alone when physical stimuli are removed. It is also interesting to note that there is a trend that the number of extremities of neurite decreased under strain treatment but only showed a significant decrease when compared with co-stimulation. The possible reason is the increased activation of RhoA GTPase by cyclic strain. Small GTPases, Rho, Rac, and Cdc42 are well-known regulators of the actin cytoskeleton and are critical for neuronal morphogenesis. The activation of RhoA GTPase will induce cell alignment perpendicular to the direction of strain (Kaunas et al., 2005; Goldyn et al., 2009) but inhibit a branch extension of neurons (Lee et al., 2000; Li et al., 2002). Leong et al. reported that Rac1, but not RhoA, activation triggered by low train at 0.5%, 0.5 Hz, was the regulator for hMSC neural differentiation (Leong et al., 2012). The function of Rac1 and RhoA in growth cone of neurons is also verified in electrical field (Rajnicek et al., 2006). Taken together, co-stimulation may cause a different balance of activities of GTPases (Rac, RhoA, Cdc42) from strain alone, under which increased RhoA activation inhibited neurite branching and finally resulted in a different morphological outcome. Moreover, this hypothesis needs to be investigated in future work.

Filopodia play important roles in neuronal branching morphogenesis, sensing the microenvironment, and formation of synaptic connections (Mattila and Lappalainen, 2008; Menna et al., 2011; Heckman and Plummer, 2013; Fischer et al., 2019). There is a marked increase in filopodia density of differentiated BMSCs with electrical stimulation and co-stimulation. This is expected, as electrical stimulation has been reported to promote neurite branching in primary neurons (Stewart et al., 2016), neural stem cells (Stewart et al., 2015), and PC12 cell lines (Manivannan and Terakawa, 1994). The filopodial sprouting strongly related with Ca^2+^ concentration and influx (Manivannan and Terakawa, 1994; Heckman and Plummer, 2013; Hu and Hsueh, 2014), and in return, filopodia increase the neurite sensitivity to stimuli. This was observed in our result (Figure 5). Strain-stimulated cells with less filopodia showed lower calcium influx in response to acetylcholine and KCl.

Co-stimulation affects not only the morphological change but also the neural gene expression. Our results show that co-stimulation significantly increased the gene expression of specific neural markers, mature neuronal marker MAP2, neuron marker β-tubulin III, and immature marker nestin. The neurotrophins, BDNF, NT-3, and NT-4 are also upregulated under co-stimulation. Neurotrophins are implicated in multiple roles in the development and function of the nervous system. BDNF plays a vital role in the survival and differentiation of MSC and neural stem cells into neurons (Trzaska et al., 2009; Chen et al., 2017; Li et al., 2017). NT-3 and NT4 were found to improve neurite growth, axonal regeneration, and functional recovery (English et al., 2005; Wu et al., 2008; Hechler et al., 2010). The gene expression level of MAP2 and NSE under co-stimulation seemed a little higher than strain or electrical stimulation alone, but there is no significant difference. Furthermore, the increase of cAMP is observed in cells under co-stimulation. Previous studies have demonstrated the effect of cAMP on neurite outgrowth, axonal growth, and neuron maturation (Cai et al., 2002; Fujioka et al., 2004; Aglah et al., 2008). Moreover, exogenous cAMP is used to induce MSC and neural stem cell differentiation into neuron cells (Deng et al., 2001; Lepski et al., 2013; Shahbazi et al., 2016). As a whole, our results indicate that cyclic strain and electrical co-stimulation can promote neural differentiation of rBMSCs.

ECM and cytoskeletal proteins are reported to be key determinants of neural growth, migration, development, function, and extension of lamellipodia (Olson and Nordheim, 2010; Broadie et al., 2011). KEGG pathway enrichment showed that focal adhesion and ECM–receptor interaction were enriched under strain and electrical co-stimulation conditions. In addition, the protein–protein interaction analysis also shows that the extracellular matrix and membrane integrin are involved in co-stimulation. The rearrangement of the cytoskeleton could activate transducers and transcriptional modulators. Previous research demonstrated that electrical stimulation could increase neurite outgrowth of PC12 cells by activating PKC to increase the NGF-induced phosphorylation of ERK1/2 (Chang et al., 2013). GO and KEGG pathway enrichment analyses and the protein level tested by alpha screen reveal that the phosphorylation of ERK1/2 and AKT is involved in neural differentiation under cyclic strain and electrical co-stimulation. The phosphorylation of ERK1/2 and AKT under co-stimulation was notably increased than under strain and electrical stimulation alone. It is well documented that AKT can improve the survival of neurons (Jo et al., 2012; Wang et al., 2016) and improve axonal growth and branching (Grider et al., 2009), and ERK signaling can promote axonal extension (Huang et al., 2017).

**FIGURE 9:**
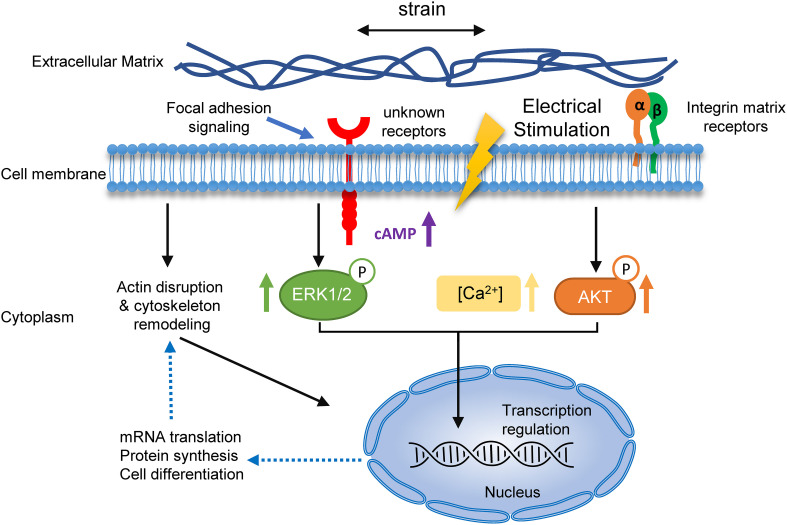
A hypothetical mechanism for the role of cyclic strain and electrical co-stimulation in promoted BMSCs neural differentiation. Schematic summary of the mechanism of strain and EF co-stimulation-induced BMSC neural differentiation. The strain and EF cause changes of ECM, which are sensed by specific receptors and integrins on the cell membrane, resulting in receptor-mediated cell mechanosensing. Activation of these receptors leads to a series of events, including actin disruption and cytoskeleton remodeling, rising of calcium and cAMP, and phosphorylation of ERK and AKT. Signals transduce into the nucleolus and then regulate neural differentiation-related mRNA transcription and protein synthesis, and in return regulate actin formation in neurites.

Based on our findings and previous studies, a putative mechanism of cyclic strain and electrical co-stimulated BMSC neural differentiation is proposed (Figure 9). Under stretch and electrical stimulation, integrins or other membrane receptors detect the change of ECM and then regulate the remodeling of cytoskeleton and increase the cAMP level and activation of certain signaling pathways (such as Ca^2+^ increasing and phosphorylation of ERK and AKT). Then, the signals activate transcription factors to regulate the transcription of neural differentiation genes. Subsequently, neural marker and neurotrophin expressions increase and then regulate actin formation in return, promoting neurite outgrowth and branching. Further molecular experiments are needed to be conducted to discover precise mechanisms of EF and stretch synergetic effects on BMSC neural differentiation.

## Conclusion

Our findings demonstrate that cyclic strain and electrical co-stimulation have a synergetic effect on EGF and FGF2-induced rat BMSC neural differentiation by upregulating neural markers and neurotropic mediators and increase calcium influx, intracellular cAMP, and phosphorylation of ERK1/2 and AKT. Knowledge of the impact of this strain and electrical co-stimulation on BMSC differentiation provides a better understanding on how cells respond to biomechanical manipulations and suggests new approaches for stem cell neural differentiation.

## Data Availability Statement

The datasets presented in this study can be found in online repositories. The names of the repository/repositories and accession number(s) can be found below: NCBI BioProject, accession no: PRJNA666744.

## Ethics Statement

The animal study was reviewed and approved by the Beihang University.

## Author Contributions

HC planned and carried out the experiments, performed the data analyses and interpretation of the results, and wrote the manuscript. YH participated in the planning of the experiments and the revision of the manuscript. WC contributed to the bulk RNA-seq data analysis. JC designed the device and took some of the confocal images. TL, JN, and RW contributed to sample preparation. YH and YF supervised and administered the project. All authors read and approved the final manuscript.

## Conflict of Interest

The authors declare that the research was conducted in the absence of any commercial or financial relationships that could be construed as a potential conflict of interest.

## Abbreviations

BMSC: bone marrow-derived mesenchymal stem cells
EF: electrical field
EGF: epidermal growth factor
FGF2: fibroblast growth factor 2
cAMP: cyclic AMP
KCl: potassium chloride
ERK: extracellular-signal-regulated kinase
AKT: protein kinase B
DMEM-LG: Dulbecco’s modified Eagle medium-low glucose
NSE: neuron-specific enolase
MAP2: microtubule-associated protein 2
NT-3: neurotrophin 3
NT-4: neurotrophin 4
BDNF: brain-derived neurotrophic factorl
GAPDH: glyceraldehyde-3-phosphate dehydrogenase.
