# Supplementary material for: Cyclic Strain and Electrical Co-stimulation Improve Neural Differentiation of Marrow-Derived Mesenchymal Stem Cells
Source: Front Cell Dev Biol. 2021 May 11;9:624755. doi: 10.3389/fcell.2021.624755 (PMC8150581; doi:10.3389/fcell.2021.624755)
Supplement: Supplementary file 1 [file Data_Sheet_1.PDF]

Supplementary Material

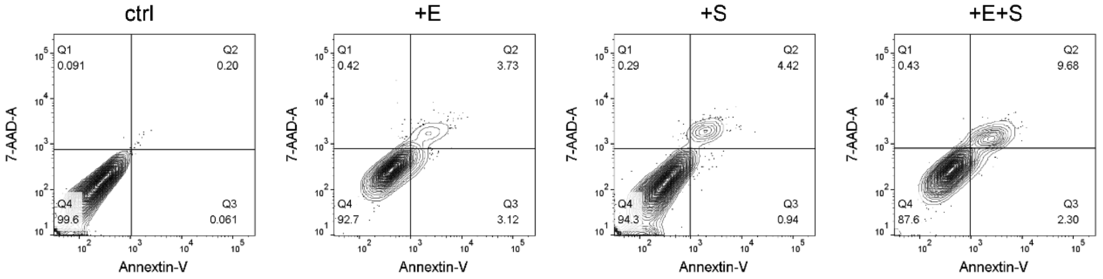

Supplementary Figure 1. Effect of strain and electrical stimulation on cell viability. PE Annexin-V Apoptosis Detection Kit I (BD Pharmingen™, cat#559763) was used to detect cell viability. Cells are in late apoptosis or already dead are both Annexin V and 7-AAD positive.
